# Neural correlates of audiovisual integration in schizophrenia – an ERP study

**DOI:** 10.3389/fpsyt.2024.1492266

**Published:** 2024-12-10

**Authors:** A. Borgolte, C. Sinke, L. Michalke, L. Möde, N. Lepsy, D. Wiswede, S. Bleich, G. R. Szycik, E. Ghaneirad

**Affiliations:** ^1^ Department of Psychiatry, Social Psychiatry and Psychotherapy, Hannover Medical School, Hannover, Germany; ^2^ Division of Clinical Psychology and Sexual Medicine, Department of Psychiatry, Social Psychiatry and Psychotherapy, Hannover Medical School, Hannover, Germany; ^3^ Department of Neurology, University of Lübeck, Lübeck, Germany; ^4^ Center for Systems Neuroscience, University of Veterinary Medicine Hannover, Hanover, Germany

**Keywords:** schizophrenia, ERP, audiovisual integration, simultaneity judgement, EEG, multisensory perception

## Abstract

**Introduction:**

Multisensory integration (MSI) enhances perception by combining information from different sensory modalities. In schizophrenia, individuals often exhibit impaired audiovisual processing, resulting in broader temporal binding windows (TBWs) which appear to be associated with symptom severity. Since the underlying mechanisms of these aberrations are not yet fully understood, the present study aims to investigate multisensory processing in schizophrenia in more detail.

**Methods:**

Individuals with schizophrenia (SZ) and healthy controls (HC) performed a simultaneity judgement task, a paradigm that is suitable for the examination of multisensory integration processes. The paradigm was also conducted to allow for the comparison of perceptions under ecologically valid and invalid conditions. Additionally, EEG recordings were made to explore underlying neural mechanisms.

**Results:**

In line with previous research, we replicated enlarged TBWs in SZ compared to HC, independent of ecological validity. Neurophysiological data further revealed reduced amplitudes in the early ERP complex N1/P2 in SZ compared to HC.

**Discussion:**

Since amplitude reduction in the N1/P2 complex is often associated with audiovisual integration processes, the results highlight perceptual dysfunction in SZ, particularly concerning the disengagement of auditory and visual stimuli.

## Introduction

Multisensory integration (MSI) is the ability to process and combine information from different sensory modalities into a unified percept. It is a fundamental ability of human perception as it leads to improved stimulus detection and localization, as evidenced by shortened reaction times (1), which provides an evolutionary advantage by allowing earlier detection of environmental stimuli (2). Even though MSI is a crucial ability of the human brain, underlying mechanisms are not yet fully understood (3–6).

Multimodal illusions provide insight into MSI processes. One of the most extensively studied examples is the McGurk effect in which the presentation of different visual (lip movements, *‘ga’*) and auditory (speech, *‘ba’*) speech information leads to the awareness of a new percept *(‘da’*) (7). Since its discovery, the McGurk effect has been replicated and explored across a range of linguistic, cultural, and developmental contexts, offering important insights into how the brain resolves sensory conflicts (8–10). Moreover, research has shown individual differences in susceptibility to the effect, such as those associated with age, cognitive factors, or specific neurological conditions (11–13). The McGurk effect thus highlights the brain’s tendency to integrate conflicting sensory information into a coherent percept, which is a fundamental aspect of MSI.

Another illusion-based paradigm that reveals further dimensions of MSI is the sound-induced flash illusion (SIFI) (14). Here, one flash is presented with two short beep sounds with varying stimulus onset asynchronies (SOAs) between the beep sounds. This often results in the perception of two flashes with probability of illusory perception declining with increasing intervals between the two beep sounds (14, 15). This paradigm illustrates the importance of temporal proximity between stimuli for MSI.

The relevance of this temporal proximity is reflected in the temporal binding window (TBW). The TBW illustrates the temporal window in which integration of stimuli from different modalities becomes more likely (1, 16). For audiovisual stimuli, the TBW is supposed to have a bimodal width of about 200ms (16) indicating that only stimuli within this time window can be effectively integrated. This temporal width is particularly important for complex stimuli like speech, as a more flexible and broader integration is required to capture the complex properties of such stimuli. Thus, the TBW is wider for complex stimuli than for simple stimuli like light flashes and beeps (17). Additionally, the TBW differs interindividually and changes across the life span with a narrowing TBW from childhood into adulthood and a broadening TBW in older age (18).

The width of the TBW also plays an important role in different neurodevelopmental and psychiatric disorders. For example, children with autism spectrum disorders integrate audiovisual information over a longer period of time (larger TBW) which is associated with a reduced perceptional validity (19, 20). Another subgroup of patients with altered audiovisual perception are individuals with schizophrenia (SZ). Schizophrenia is a complex mental disorder affecting essential aspects of psychological experience, marked by characteristic disturbances in thinking, perception, self-functions, affectivity, drive, and psychomotor activity (21). Positive symptoms include persistent hallucinations and delusions, while negative symptoms involve blunted affect and psychomotor disturbances. Neuropsychological deficits, particularly in early stages, are a core feature of SZ, affecting attention, memory, and executive functions (22). Meta-analyses indicate significant impairments in overall cognition and specific domains such as processing speed and verbal memory (23–25).

Individuals with SZ exhibit specific functional deficits in auditory and visual perception, often experiencing auditory or visual hallucinations, which impair social communication (26–28). These perceptual impairments highlight the importance of understanding sensory information processing in SZ (29, 30). Accordingly, recent studies have identified impairments in unimodal visual perception in SZ, such as in contrast threshold recognition or temporal order discrimination (31, 32). Further studies have also indicated impairments in unimodal auditory processing in SZ (5, 33, 34). Conversely, review articles also report intact unisensory processing in SZ while multisensory processing is impaired, even with very simple, low-threshold stimuli (35). Intact unisensory processing in SZ, coupled with impaired audiovisual integration (AVI), was demonstrated, for instance, in a language-based study comparing healthy controls (HC) with SZ (33, 36). In this study, SZ did not differ from HC in unimodal auditory processing (sound localization). However, SZ benefited less from the additional visual component (lip-reading) when processing audiovisual stimuli (speech), indicating an integration deficit in audiovisual processing with complex stimuli in SZ.

As outlined above, as audiovisual integration depends on the temporal proximity of the unimodal stimuli, it can also be depicted through TBW in behavioral experiments. Many studies demonstrate that individuals with SZ have a broader TBW (i.e. integration over longer SOAs) compared to HC (37–39). This aberration is often associated with typical symptoms of schizophrenia concerning sensory dysfunction as for example the severity of hallucinations (5). However, little is known about the underlying mechanisms that account for this enlargement. For an improved understanding of audiovisual integration in SZ the exploration of underlying neural mechanisms seems promising.

EEG studies provide insights into the temporal dynamics of audiovisual integration. The N1 component, a negative deflection at about 100 ms post-stimulus, is sensitive to stimulus features and attentional processes (40). The P2 component, a positive deflection at about 200 ms post-stimulus, is associated with later stages of sensory processing, including attentional allocation and sensory integration (41).

For SZ event-related potential (ERP) studies revealed aberrant short-latency ERPs in response to the McGurk illusion compared to HC (42). Specifically, different studies involving HC as well as SZ have linked the amplitudes of early evoked N1 and P2 components to audiovisual processing (43–46). However, ERP-studies investigating audiovisual integration in SZ report inconsistent findings (47). Stekelenburg et al. (48) compared N1/P2 suppression effects in SZ and HC for auditory vs. audiovisual speech stimuli, reporting a lack of audiovisual N1 suppression and a diminished congruency effect of P2 amplitude in the SZ group. Other studies found similar effects regarding N1 suppression (49, 50). However, Wynn et al. (46) did not find any group differences for audiovisual integration at the neural level in a multisensory target detection task.

In summary, the current state of research on audiovisual integration processes in SZ remains highly inconsistent. This is likely due to the strong variation in utilized stimuli, ranging from simple to complex. Therefore, further systematic investigations of audiovisual integration processes in SZ and its underlying mechanisms are necessary.

In the presented study, we employed a simple paradigm for audiovisual simultaneity perception while recording EEG data, since such paradigms are highly useful for evaluating audiovisual integration processes (51, 52). Specifically, we use a simultaneity judgment task that includes ecological valid and invalid conditions. Ecological valid conditions comprise trials in which a beep sound is preceded by a flash while ecological invalid conditions comprise trials in which a flash follows a beep sound. This ecological valid condition reflects everyday life experience in which the light, travelling with ~300000000m/s, is the leading stimulus while the sound follows, traveling only with ~340m/s (53, 54). Therefore, people show a higher tolerance of lagging auditory than visual information in simultaneity judgement tasks (52, 55). This approach allows for the identification of group differences in TBW as a function of ecological validity. Moreover, we expand the existing literature on the relationship between early ERPs and audiovisual perceptual processes in SZ. Based on the current literature, we anticipate to replicate findings of a widened TBW in SZ compared to HC as well as altered amplitudes in early ERP components.

## Methods

### Subjects

23 subjects with SZ participated in this study. 22 HCs were matched for age and gender. Participants with SZ were recruited from psychiatric wards of Hannover Medical School. The diagnosis of schizophrenia was conducted by experienced physicians according to DSM-IV-TR (56). 16 participants were diagnosed with paranoid schizophrenia, one with a schizoaffective disorder, one with an acute polymorphic psychotic disorder without symptoms of schizophrenia and one with an acute schizophrenia-like psychotic disorder. Diagnosis was further validated using the Positive and Negative Syndrome Scale (PANSS; 57; German adaptation by 58). All SZ subjects took antipsychotic medication and were in a stable state of health when they participated in this study. The mean of the chlorpromazinequivalent was M = 330.46 (SD = 359.85). Despite the heterogeneity among SZ participants, research demonstrates similar findings regarding the TBW in this population, regardless of the state of the illness (39). Participants in the control group had no history of diagnosed psychiatric disorders. Since four subjects had to be excluded due to missing data or a lack of variance in response pattern, a total of 22 HC and 19 SZ subjects were analyzed. All participants reported normal hearing and normal or corrected-to-normal vision. Additionally age and gender, years of education as well as verbal IQ were assessed. For the assessment of verbal IQ the MWT-B (“Mehrfachwahl-Wortschatz Intelligenztest”) (59) was used. The ethics committee of Hannover Medical School approved the study (ethics approval number: 3237-2016) and all participants gave written informed consent. Detailed sample characteristics are provided in **Table 1**.

**Table 1:** Sample characteristics.

	*HC*		*SZ*		
Characteristics	*M*	*SD*	*M*	*SD*	
*N* (female/male)	22 (12/10)		19 (9/10)		χ2(1) = 0.21, p = 0.65
Age in years	38.5	12.86	40.6	12.64	*t(*39) = -0.53, *p* = 0.60
Verbal IQ	106.33	4.03	99.87	8.52	*t*(28) = 2.66, *p* = 0.01
PANSS (total)			51.28	11.50	
Positive			12.11	4.31	
Negative			12.78	5.95	

### Materials and methods

**Figure 1:**
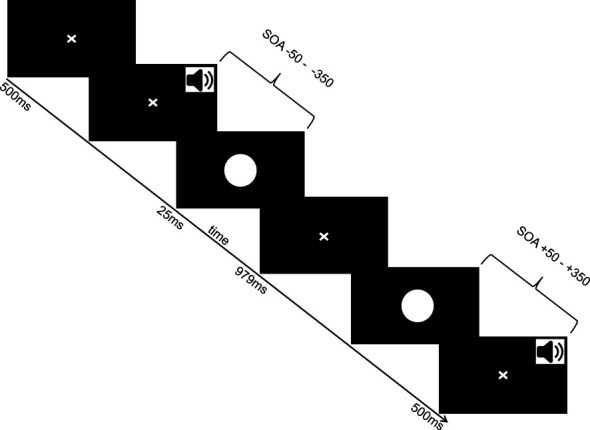
Paradigm. Visual flash and auditory beep sound were presented in varying stimulus onset asynchronies (SOAs) including ecologoically valid and invalid conditions.

Participants were seated in a soundproof chamber facing a screen fitted with two adjacent sound speakers presenting auditory information in mono mode. Visual stimuli were presented on a computer monitor (21’ Sony Trinitron Multiscan G520, Sony Electronics Inc., San Diego, CA, United States, with 1,024 × 768-pixel resolution) situated approximately 60 cm in front of the participants. Throughout the experiment, subjects were exposed to a white disk presented on the monitor and brief beep sounds with varying onset intervals between them. Their task was to determine whether the visual and auditory stimuli were presented simultaneously by pressing a designated button on the keyboard. The experimental procedure is illustrated in **Figure 1**. Stimulus delivery was managed using Presentation^©^ software. Auditory stimuli consisted of a 7ms sine wave tone at 1850 Hz. The visual stimulus, centrally displayed, covered a visual angle of roughly 6° in both dimensions. The onset asynchrony between the visual and auditory stimulus ranged from -350ms (visual following auditory stimulus) to +350ms (auditory following visual stimulus) in 50ms increments (e.g., -350, -300, -250, -200, -150, -100, -50, 0, 50, 100, 150, 200, 250, 300, 350 ms). A trial began with a fixation cross appearing at the center of the screen for 500ms, followed by the brief presentation of the visual stimulus for 25ms (perceived as a rapid flash), and another fixation cross display for 979ms. Each onset condition was presented 15 times in a pseudorandomized order, resulting in 225 trials overall. Participants were instructed to respond only upon cue and their response was followed by another 500ms fixation cross display. The experimental procedure lasted approximately 11 minutes and was part of a series of experiments exploring audiovisual integration.

### EEG acquisition and analyses

Seven subjects (5 HC and 2 SZ) had to be excluded from EEG analyses due to strong artifacts in the EEG signal. Thus, a total of 17 subjects per group were included in EEG analyses. EEG activity was captured from 32 Ag/AgCl electrodes according to international 10-20 system with a sampling rate of 512 Hz. Signals were recorded from DC using a BioSemi active electrode system (BioSemi B.V., Amsterdam, Netherlands) and the accompanying ActiView software package. Instead of reference and ground with two additional electrodes, it uses a CMS/DLR feedback loop (for more information see: http://www.biosemi.com/faq/cmsanddrl.htm). To reduce artifacts, impedances of the single electrodes were kept below 25 kΩ. The software provides estimations of eye movement artifacts from the frontocentral scalp electrodes (FP1, FP2). BESA Research 6.0 (BESA GmbH, 2013) was used for processing EEG data. Raw data was filtered with a 0.1 Hz high-pass filter, a 30 Hz low-pass filter, and a 50 Hz notch filter. Blink artifacts were corrected by applying an independent component analysis (ICA) on single subject data (60). For each subject, one component was identified and removed. If blink artifacts were still present, the ICA was repeated. EEG data segmentation into event-related epochs of 1100 ms was conducted, with the baseline starting 100 ms before stimulus onset. ERPs were time-locked to the presentation of the second stimulus. According to visual inspection of the data, N1 was defined as the maximum negative peak in the time window of 100 - 200 ms after stimulus onset, and P2 as the maximum positive peak in the time window of 150 - 275 ms after stimulus onset (41). The P2 component thus follows the N1 component and is highly associated with the N1 which is mainly generated in auditory cortical areas and reaches its maximum typically at frontocentral electrodes (61). Also existing literature finds reports relevant differences between SZ and HC in these ERP components at frontocentral scalp sites (45, 48, 50). Since the visual inspection of our data is in line with previous research, we decided on analyzing frontocentral electrodes Cz and Fz.

### Analysis

#### Behavioral data

Analysis of behavioral data was based on the percentage of reported simultaneous events. Effects were examined using univariate repeated measures analyses of variance (rm-ANOVAs) followed by *post hoc* t-tests with Bonferroni correction for multiple comparisons. When the assumption of sphericity was violated, Huynh-Feldt correction was used.

We conducted a 2x7x2 rm-ANOVA with the factors ecological validity with negative SOAs representing the ecological invalid condition and positive SOAs representing the ecological valid condition, SOA (50ms, 100ms, 150ms, 200ms, 250ms, 300ms, 350ms) and group (SZ and HC). The condition with a SOA of 0ms was not entered into analysis but used as a control condition to assure compliance.

To estimate a temporal binding window as well as the point of subjective simultaneity for both groups, a Gaussian distribution was fitted to the individual mean data using MATLAB software (Version R2023a, The MathWorks Inc., Natick,MA, USA),. SZ and HC were fitted separately, using the nonlinear least square method (62) with mean, standard deviation (SD) and amplitude as free parameters (mean R² =0.78, with mean R² = 0.85 for HC and mean R² = 0.69 for SZ). The PSS was defined as the mean of the fitted Gaussian while the TBW was defined as SD. Size of the TBW and PSS were compared between groups via t-test.

#### EEG data

For the EEG data, the stimulus onset asynchronies were collapsed prior to analyses to ensure sufficient statistical power, as movement artifacts led to a reduction in the number of available trials. We computed the size of individualized temporal binding windows for each subject by determining the standard deviation around the individual PSS. All SOA conditions (stimulus onset asynchrony) that fell within one standard deviation (after rounding) around the PSS were grouped into SOA conditions within the temporal binding window, while the remaining SOA conditions were grouped into those outside the temporal binding window. For negative SOA conditions in HC, on average, conditions up to SOA 150 were grouped into the SOA conditions within the temporal binding window (M=2.94, SD = 1.1, Min=1, Max=5). For positive SOA conditions in HC, on average, conditions up to SOA 100 were grouped into the SOA conditions within the temporal binding window (M=2.35, SD = 1.3, Min=1, Max=6). For negative SOA conditions in SZ, on average, conditions up to SOA 200 were grouped into the SOA conditions within the temporal binding window (M=2.94, SD = 1.6, Min=2, Max=7). For positive SOA conditions in SZ, on average, conditions up to SOA 200 were grouped into the SOA conditions within the temporal binding window (M=4.37, SD = 1.4, Min=2, Max=7). As for SZ, the standard deviation for three subjects was so large that no conditions could be included in the condition outside the temporal binding window, which is consistent with previous research (37). Consequently, these subjects were excluded from the analyses.

Three separate rm-ANOVAs for N1, P2 and N1/P2-complex were conducted. The N1/P2-complex was defined as the difference between P2 and N1 values. The 2x2x2x2 rm-ANOVAs included the factors ecological validity (positive and negative), temporal binding window (inside and outside), electrode (Cz and Fz) and group (HC and SZ). In line with the analyses of behavioral data, the rm-ANOVAs were followed by *post hoc* t-tests with Bonferroni correction for multiple comparisons.

## Results

### Behavioral results

**Figure 2:**
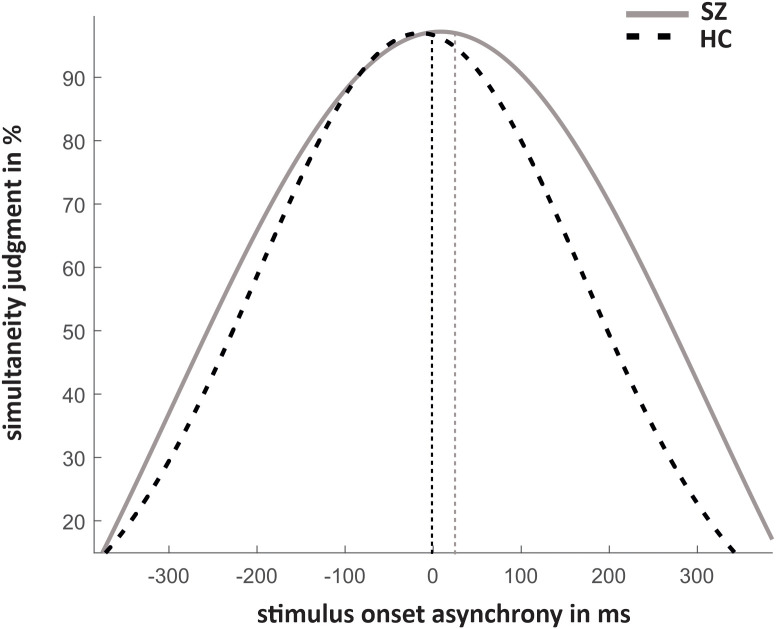
Mean simultaneity judgments in % and standard errors for each SOA condition for SZ and HC with negative SOAs representing ecologically invalid conditions and positive SOAs ecologically valid conditions.

**Figure 3:**
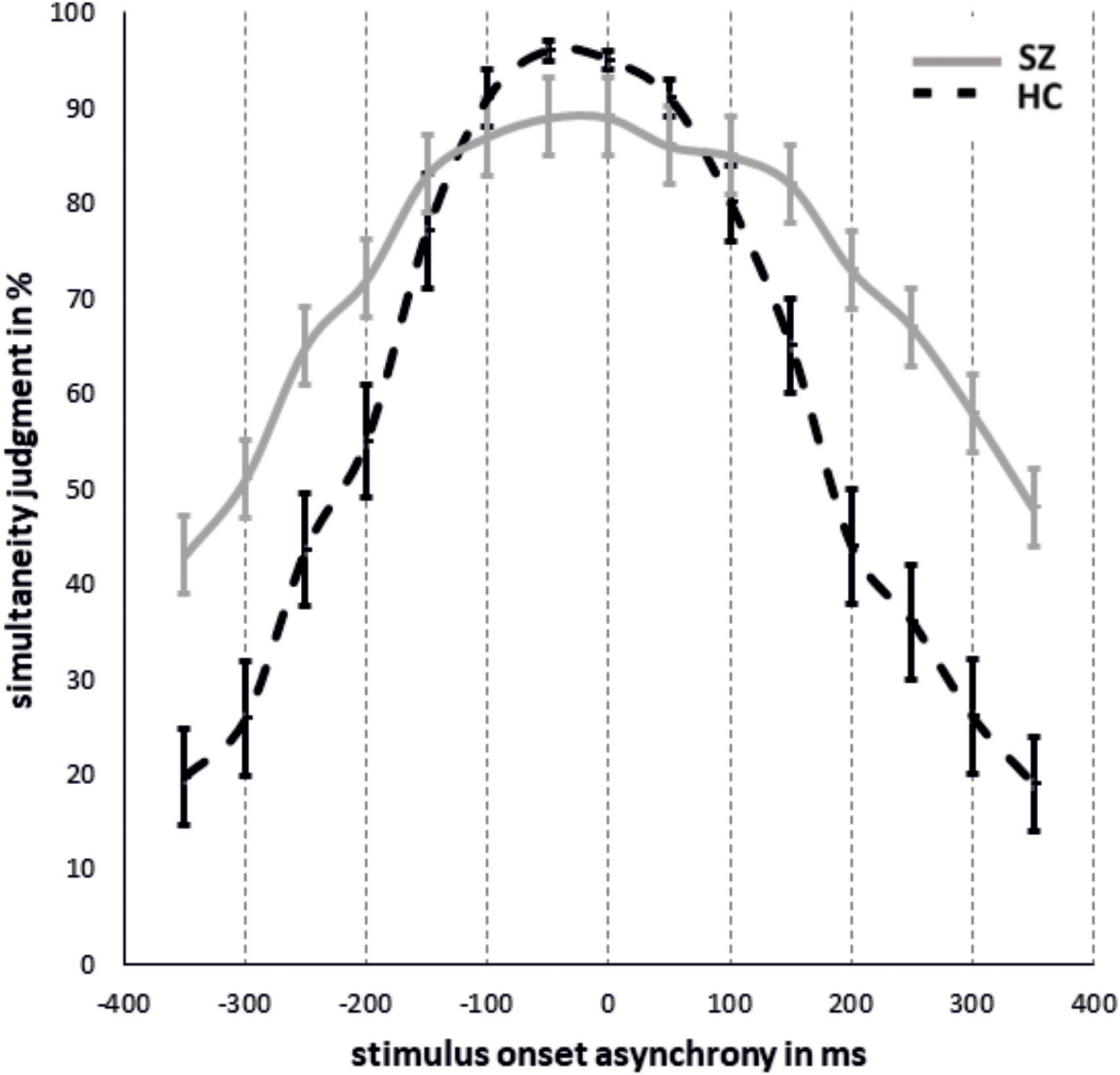
Mean fitted Gaussian distribution for both groups to estimate the ‘Point of Subjective Simultaneity’.

The results of simultaneity judgments and the fitted data are shown in **Figures 2**, **3**. Significant group differences did not emerge for simultaneous presentations (SOA_0_: T_(26,22)_ = 1,63, p = 0.12).

The rm-ANOVA revealed a main effect of SOA (F_(3.41,133.10)_ = 133.33, p < 0.001, Huynh-Feld corrected, η^2^ = 0.77) and a main effect of group (F_(1,39)_ = 8.54, p = 0.006, η^2^ = 0.18) that were further characterized by a significant SOA*group interaction (F_(3.41,39)_ = 12.96, p < 0.001, Huynh-Feld corrected, η^2^ = 0.25). *Post-hoc* t-tests showed significantly higher rates of simultaneity judgment for SZ compared to HC in SOA conditions 350ms (p < 0.001), 300ms (p < 0.001), 250ms (p < 0.001) and 200ms (p = 0.006). For other SOAs no group differences were observed. No other main effects or interactions reached significance. However, a trend for significance was observed for the group*ecological validity interaction (F_(1,39)_ = 3.37, p = 0.07, η^2^ = 0.08) with significantly more simultaneity judgements for negative SOAs (ecologically invalid condition) in HC compared to SZ (p = 0.04). No perceptual differences were found between ecological validity conditions in SZ (p > 0.1). Due to a limited power, we also decided on repeating the rm-ANOVA for groups separately. For HC we find a significant main effect of ecological validity (F_(1,21)_ = 5.44, p = 0.03, Huynh-Feldt corrected, η^2^ = 0.21) and a significant main effect of SOA (F_(3.63,76.14)_ = 117.99, p < 0.001, Huynh-Feldt corrected, η^2^ = 0.85). *Post-hoc* tests revealed significantly higher rates of simultaneity perceptions in negative SOA conditions compared to positive SOA conditions (p = 0.03). *Post-hoc* tests for SOA conditions are reported in **Table 2**.

**Table 2:** P-values for *post-hoc* t-tests comparing SOA conditions in HC.

SOA in ms	*± 100*	*± 150*	*±* 200	*±* 250	*±* 300	*±* 350
*± 50*	0.13	**<0.001**	**<0.001**	**<0.001**	**<0.001**	**<0.001**
*± 100*		**0.001**	**<0.001**	**<0.001**	**<0.001**	**<0.001**
*± 150*			**0.005**	**<0.001**	**<0.001**	**<0.001**
*± 200*				**<0.001**	**<0.001**	**<0.001**
*± 250*					**0.004**	**0.002**
*± 300*						0.18

Bold values indicate significant differences (p < 0.01).

For SZ we find a significant main effect of SOA (F_(2.7,49.21)_ = 33.10, p < 0.001, Huynh-Feldt corrected, η^2^ = 0.65). *Post-hoc* tests for SOA conditions are reported in **Table 3**. No other main effects or interactions reached significance.

**Table 3:** P-values for *post-hoc* t-tests comparing SOA conditions in SZ.

SOA in ms	*± 100*	*± 150*	*±* 200	*±* 250	*±* 300	*±* 350
*± 50*	0.11	**<0.001**	**<0.001**	**<0.001**	**<0.001**	**<0.001**
*± 100*		**0.02**	**0.02**	**<0.001**	**<0.001**	**<0.001**
*± 150*			**1**	**0.006**	**0.002**	**0.005**
*± 200*				**0.02**	**0.01**	**0.02**
*± 250*					0.64	0.54
*± 300*						1

Bold values indicate significant differences (p < 0.01).

The PSS did not differ between groups (HC: -3.80 ± 51.74, SZ: 21.22 ± 120.13; T(_23.71_)=-0.84, p = 0.41). The TWS was significantly larger for SZ compared to HC (HC: 323.57 ± 149.57; SZ: 460.22 ± 175.29; T(_38_) = -2.661, p = 0.01).

### EEG results

#### P2

**Figure 4:**
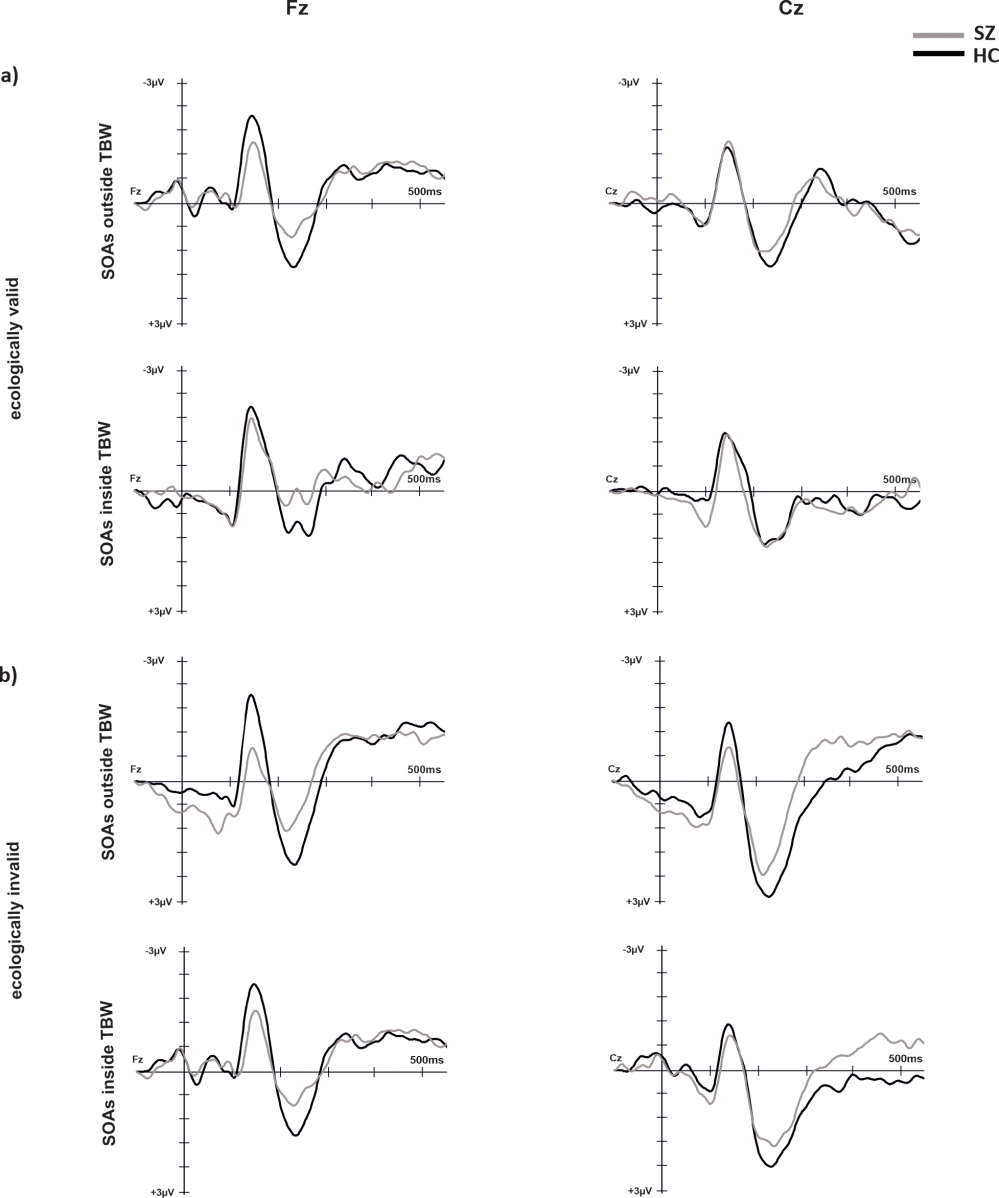
ERPs over electrode positions Fz and Cz for SZ and HC under ecologically valid **(A)** and invalid **(B)** conditions.

**Figure 5:**
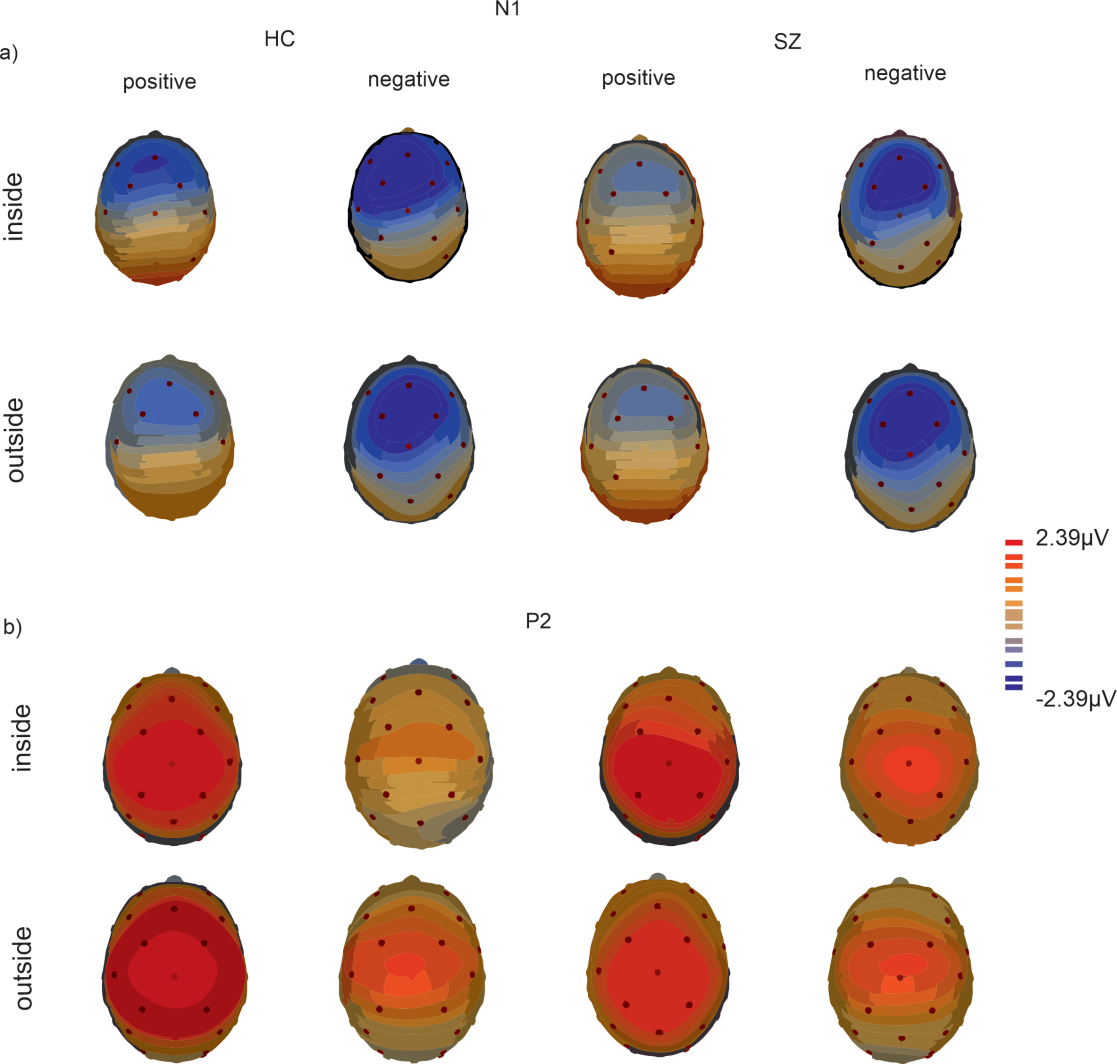
Topographical maps depict the scalp distribution at 150 ms for N1 **(A)** and 212 ms for P2 **(B)** over all SOA conditions inside and outside the temporal binding window for HC and SZ.

The results of electrophysiological data are presented in **Figures 4**, **5**. For P2 the main effect of electrode (F(_1, 29_)=11.76, p = 0.002, η^2^ = 0.29) reached significance with lower amplitudes for Fz compared to Cz (p=0.002, M_Diff_ = 1.30, 95%-CI[0.52, 2.07]). No other main effects or interactions reached significance.

#### N1

For N1 the main effect of temporal binding window (F(_1, 29_)=4.59, p = 0.04, η^2^ = 0.14) reached significance with more negative amplitudes for SOAs inside the temporal binding window compared to outside the temporal binding window (p=0.04, M_Diff_ = 0.46, 95%-CI[0.02, 0.78]). Moreover, the main effects of validity (F(_1, 29_)=7.60, p = 0.01, η^2^ = 0.21) and electrode (F(_1, 29_)=25.76, p < 0.001, η^2^ = 0.47) reached significance with more negative amplitudes at electrode Fz compared to Cz (M_Diff_ = 0.77, 95%-CI[0.46, 1.08]). The main effect of validity was further characterized by a significant interaction of validity and group (F(_1, 29_)=4.59, p = 0.04, η^2^ = 0.14) with *post-hoc* tests revealing significant group differences only for positive SOAs (p=0.02, M_Diff_ = 1.11, 95%-CI[0.17, 2.05]) with more negative amplitudes for HC compared to SZ.

#### N1/P2

For the N1/P2-complex, the main effect of group reached significance (F(_1, 29_)=5.49, p = 0.03, η^2^ = 0.16) with a larger complex for HC compared to SZ. No other main effects or interactions yielded significance.

To investigate possible connections between the amplitudes of the early event-related potentials, we performed a Pearson-correlation analysis for groups separately. Corresponding to the reported results, at electrode Fz, we calculated a correlational analysis for N1 and the N1/P2-complex values in each SOA condition inside the TBW with the averaged simultaneity judgments in each SOA condition inside the TBW. Based on previous studies, we expected to find correlations between simultaneity judgments and the amplitude of the N1 as well as the N1/P2 (45, 63). Consequently, a Bonferroni correction for multiple comparisons was applied across three tests, resulting in an adjusted p-value of 0.025.

No significant correlations were observed for the N1 or the N1/P2 complex, for neither HC nor SZ (all p > 0.025).

Finally, we wanted to assess for possible relations between temporal integration and symptom severity in SZ. Therefore, we performed a Pearson-correlation analysis between PANSS total score and size of the TBW. The analyses revealed a significant positive correlation (r = 0.61; p = 0.02) implicating that a higher symptom severity is associated with a larger TBW. Additionally, PANSS total score and amplitudes of early event-related potentials (N1-amplitudes as well as the N1/P2 complex) were correlated. Again, a Bonferroni correction for multiple comparisons was applied across three tests, resulting in an adjusted p-value of 0.025. No significant correlations were observed.

## Discussion

In the present study, we investigated audiovisual integration in patients with schizophrenia (SZ) compared to healthy controls (HC) using a simultaneity judgment paradigm. During the behavioral experiment, electrophysiological data were collected via EEG, allowing for a comparison of early ERP components between the groups.

First, regarding the Gaussian distribution that was fitted to our behavioral data, we found a sufficient goodness of fit for both groups (mean R² = 0.78, with mean R² = 0.85 for HC and mean R² = 0.69 for SZ) (64). This is in line with previous literature demonstrating a Gaussian or sigmoid distribution for simultaneity judgement tasks (65–67). Moreover, for the control condition (simultaneous presentation of flash and beep) we did not find any group differences, indicating that groups did not differ systematically in response bias. These findings show that our paradigm was sufficient to evoke the intended results.

One objective of the present study was to replicate findings from previous studies and demonstrate differences in temporal aspects of audiovisual perception for individuals with SZ compared to HC (47). Significant differences were found between the groups in the perception of simultaneity of the presented audio and visual stimuli for certain SOA conditions. Specifically, SZ perceived stimuli as simultaneous significantly more often at long SOA conditions (200ms to 350ms) compared to HC. These findings indicate an impaired ability to separate audiovisual stimuli in SZ compared to HC, which is consistent with earlier studies that found alterations in the perception of audiovisual stimuli in SZ at longer SOAs (37, 39, 68). The point of subjective simultaneity (PSS) did not differ between groups, however, the temporal binding window for SZ was significantly larger than for HC. This indicates that SZ perceive asynchronously presented audiovisual stimuli as belonging together over a longer period of time compared to HC. Our findings also support previous research reporting a broader temporal window for integrating audiovisual stimuli in SZ (17, 37, 39). Additionally, we observed a positive correlation between symptom severity and width of the TBW in SZ, which is in line with research reporting associations between deficits in multisensory temporal processing and aberrations in sensory perception (5).

Interestingly, we found a trend for significance for the interaction of ecological validity and group. The presented results suggest that HC have greater difficulty separating audiovisual stimuli under ecologically invalid conditions compared to valid conditions. No such difference is observed in individuals with SZ. It is likely that this potential difference for HC reflects our everyday experiences of differences in physical characteristic of stimuli (light is mostly faster than sound). Therefore, one might assume that the perceptual system should be more effective in separating or integrating stimuli that correspond to the natural environmental perception, and thus in the ecologically valid condition. In the ecologically invalid condition, however, we cannot rely on perceptual experience and unconscious learning processes, making differentiated perception more difficult.

No such difference is observed in individuals with SZ. The lack of difference between ecologically valid and invalid conditions might imply an aberration of perceptual experience in SZ. Correspondingly, our findings might support the application of the predictive coding model as a useful framework for understanding multisensory integration deficits observed in SZ (69, 70). Predictive coding theory posits that the brain continuously anticipates incoming sensory information based on an internal model derived from learned statistical regularity in sensory experience. This model is refined through a hierarchical, bidirectional signaling system: top-down predictions, informed by prior knowledge, to account for sensory inputs, while bottom-up prediction errors are generated to adjust the internal model when discrepancies arise (71, 72). Previous studies have provided evidence for predictive mechanisms in audiovisual speech processing (e.g., 73–76), yet few have focused on simple stimulus synchronicity, as used in our paradigm. Here, we explored ecologically valid conditions where visual stimuli precede auditory stimuli, aligning with typical sensory experiences. According to predictive coding, visual stimuli would facilitate the prediction of corresponding auditory inputs via top-down signaling. Conversely, in invalid conditions, an unexpected sequence would likely trigger a feedforward signal to update the internal model due to prediction error. Our data indicate that HC participants show notable differences between valid and invalid conditions, likely due to effective predictive mechanisms. However, SZ participants displayed no differences, possibly reflecting an imbalance in predictive precision. Recent research suggests that SZ involves an abnormal weighting between the precision of predictions and sensory inputs, with SZ associated with either a reduced precision of prior beliefs or an increased precision of sensory data. This imbalance may shift perceptual processing towards sensory inputs rather than prior expectations, potentially leading to aberrant salience of sensory information and contributing to the atypical multisensory integration observed in SZ.

To further elucidate relevant processes of multimodal perception examining neural correlates is a promising account. For audiovisual integration processes, a connection between early event-related potentials N1 and P2 has consistently been reported (43, 77, 78). Stekelenburg and Vroomen (78) used ecologically valid auditory and visual stimuli to examine audiovisual integration processes. They found reduced auditory N1 amplitudes for audiovisual stimuli indicating facilitated processing for multisensory stimuli. Similar effects of amplitude suppression were found for the P2 component when examining speech-related stimuli (43). The N1 component is highly associated with auditory perception and is involved in early sensory processing, while the P2 component is associated with cognitive processes related to sensory processing such as attention allocation (79). However, the ERP components may reflect different neural mechanisms dependent on stimulus features and task requirements (79, 80). In the present study, we found significant differences between SZ and HC in neurophysiological data. In line with Stekelenburg and Vroomen (81), the N1 component was significantly smaller in valid conditions for SZ compared to HC. Regarding the N1/P2-complex, we find reduced amplitudes for SZ compared to HC for both validity conditions. These results can be interpreted in line with previous research regarding audiovisual integration since an amplitude reduction in the N1/P2 complex is associated with audiovisual integration processes (43, 77, 78). This interpretation is in line with behavioral data showing an enlarged TBW for SZ compared to HC indicating integrational processes over a longer period.

However, some of our results contrast with previous findings challenging the aforementioned interpretation. First, we find a significantly larger N1 amplitude for SOA conditions inside the TBW. As mentioned before, previous research consistently reported a N1 suppression associated with audiovisual integration (45, 46, 78). Therefore, a reduced N1 amplitude would be expected for those SOAs inside the TBW compared to outside since audiovisual integration processes take place inside the TBW. Another surprising result is that we do not find any significant correlations between ERP amplitudes and simultaneity perception on a behavioral level or between ERP amplitudes and symptom severity in SZ. The present task required subjects to separate auditory and visual stimuli, instead of integrating them. It is possible that different mechanisms are crucial to be able to separate auditory and visual stimuli. For this reason, typical ERP changes associated with audiovisual integration processes might not have manifested, as other neural mechanisms may be involved. In this context, the N1 amplitude could reflect the increased attentional resources required to separate auditory and visual stimuli when SOA conditions are short. Accordingly, we observed enhanced N1 amplitudes for valid conditions only in HC. As outlined in the behavioral results, it is possible that, through everyday experience, HC develop mechanisms that facilitate the separation of multisensory stimuli, which is reflected in an enlarged N1 amplitude. Since invalid conditions are not typically encountered in regular experience, such mechanisms may not develop, leading to the absence of N1 enhancement in these cases. Interestingly, for SZ no differences between valid and invalid conditions were observed which may reflect a deficient ability to activate mechanisms that support separation of multisensory stimuli. This may be highly associated with typical symptoms of schizophrenia and may play a crucial role in misperceptions such as hallucinatory symptoms (5). However, this explanation is highly speculative since we did not include unisensory control conditions to ensure that reduced amplitudes in SZ are associated with audiovisual integration. In addition, as outlined above, we did not find any correlations between symptom severity and N1 amplitude.

Surprisingly, we did not observe any group differences for the isolated ERP component P2, nor did we find significant correlations between ERP components and behavioral data. Additionally, our findings regarding the N1 component differ from previous studies, which have reported reduced N1 amplitudes for short SOAs within the same simultaneity judgment paradigm (63, 82). It is important to consider differences in study design, as early ERPs are highly sensitive to specific stimulus characteristics (41). The aforementioned studies utilized different stimulus sets, often involving greater complexity; for example, Kaganovich and Schumaker (63) presented a drawing of an explosion. Furthermore, discrepancies in study samples may have contributed to the differing results, as the participants in our study were older than those in previous research, and age is known to influence multisensory integration (16). Future research should therefore emphasize the impact of specific task requirements, stimuli, and sample characteristics to deepen the understanding of MSI. Additionally, it is necessary to acknowledge the limitations of the present study.

### Limitations

An important limitation is a relatively small sample size as well as a limited number of trials. Even though sample size corresponds to similar studies (83, 84) it is possible that the power was too small for effects to be shown. Additionally, trial number was limited to ensure sufficient attentional resources of the SZ group to perform the task. Therefore, some analyses could not be performed. For example, a previous study that used a similar paradigm found differences in P2 amplitudes between trials that were perceived as simultaneous vs. non-simultaneous (82). Due to a relatively small number of trials per SOA condition as well as data loss in EEG data, in the present analyses we entered all trials irrespective of reported simultaneity perception. Thus, possible relations between simultaneity perception and ERP-components may not have been sufficiently analyzed.

Additionally, the set up of the present experiment has to be discussed. ERPs were time-locked to the presentation of the second stimulus. However, we did not synchronize termination of the stimuli. Even though it is reasonable to assume that processes of audiovisual perception are locked to the onset of the second stimulus, it cannot be excluded that these timing differences may have influenced recorded ERPs.

## Conclusion

The present study examined audiovisual simultaneity judgements under ecologically valid and invalid conditions for SZ compared to HC. We found an enlarged temporal binding window for SZ for valid as well as invalid conditions, which is in line with previous research. Regarding EEG-data, we found contradictory results. We did not find relations between amplitude of early ERPs and simultaneity perception, which is in contrast to previous studies. Additionally, we found larger N1 amplitudes for HC compared to SZ in positive SOA conditions, while overall, an N1 enhancement was observed for SOA conditions inside the TBW compared to outside, irrespective of group condition. One possible explanation may be that different neural mechanisms are involved in audiovisual separation and integration processes. However, it remains unclear what exact mechanisms may underlie audiovisual separation. More research is needed that combines tasks demanding audiovisual separation and integration. For SZ, we found reduced amplitudes in the N1/P2-complex compared to HC. These differences may be associated with stronger audiovisual integration in SZ.

All in all, it became evident that patients with SZ differ in the perception of audiovisual stimuli under both ecologically valid and invalid conditions on behavioral and neurocognitive measures. However, more research is needed to fully understand perceptional aberrancies in schizophrenia.

## Data Availability

The raw data supporting the conclusions of this article will be made available by the authors, without undue reservation.

## References

[1] Zmigrod S Hommel B. Feature integration across multimodal perception and action: A review. Multisensory Res. (2013) 26:143–57. doi: 10.1163/22134808-00002390 23713203

[2] Hershenson M. Reaction time as a measure of intersensory facilitation. J Exp Psychol. (1962) 63:289–93. doi: 10.1037/h0039516 13906889

[3] Brandwein AB Foxe JJ Butler JS Frey HP Bates JC Shulman LH. Neurophysiological indices of atypical auditory processing and multisensory integration are associated with symptom severity in autism. J Autism Dev Disord. (2015) 45:230–44. doi: 10.1007/s10803-014-2212-9 PMC4289100 25245785

[4] Choi I Demir I Oh S Lee SH. Multisensory integration in the mammalian brain: diversity and flexibility in health and disease. Philos Trans R Soc B. (2023) 378(1886):20220338. 10.1098/rstb.2022.0338 PMC10404930 37545309

[5] Stevenson RA Park S Cochran C McIntosh LG Noel J-P Barense MD. The associations between multisensory temporal processing and symptoms of schizophrenia. Schizophr Res. (2017) 179:97–103. doi: 10.1016/j.schres.2016.09.035 27746052 PMC5463449

[6] Stein BE Meredith MA Wallace MT. The visually responsive neuron and beyond: multisensory integration in cat and monkey. Prog Brain Res. (1993) 95:79–90. doi: 10.1016/S0079-6123(08)60359-3 8493355

[7] McGurk H MacDonald J. Hearing lips and seeing voices. Nature. (1976) 264:746–748. doi: 10.1038/264746a0 1012311

[8] Alsius A Navarra J Campbell R Soto-Faraco S. Audiovisual integration of speech falters under high attention demands. Curr Biol. (2005) 15:839–43. doi: 10.1016/j.cub.2005.03.046 15886102

[9] Nath AR Beauchamp MS. A neural basis for interindividual differences in the McGurk effect, a multisensory speech illusion. Neuroimage. (2012) 59:781–7. doi: 10.1016/j.neuroimage.2011.07.024 PMC3196040 21787869

[10] Sekiyama K Tohkura YI. McGurk effect in non-English listeners: Few visual effects for Japanese subjects hearing Japanese syllables of high auditory intelligibility. J Acoustical Soc America. (1991) 90:1797–805. doi: 10.1121/1.401660 1960275

[11] Altieri N Lentz JJ Townsend JT Wenger MJ. The McGurk effect: An investigation of attentional capacity employing response times. Attention Perception Psychophysics. (2016) 78:1712–27. doi: 10.3758/s13414-016-1133-4 27188651

[12] Pearl D Yodashkin-Porat D Katz N Valevski A Aizenberg D Sigler M. Differences in audiovisual integration, as measured by McGurk phenomenon, among adult and adolescent patients with schizophrenia and age-matched healthy control groups. Compr Psychiatry. (2009) 50:186–92. doi: 10.1016/j.comppsych.2008.06.004 19216897

[13] Setti A Burke KE Kenny R Newell FN. Susceptibility to a multisensory speech illusion in older persons is driven by perceptual processes. Front Psychol. (2013) 4:575. doi: 10.3389/fpsyg.2013.00575 24027544 PMC3760087

[14] Shams L Kamitani Y Shimojo S. What you see is what you hear. Nature. (2000) 408:788–8. doi: 10.1038/35048669 11130706

[15] Foss-Feig JH Kwakye LD Cascio CJ Burnette CP Kadivar H Stone WL. An extended multisensory temporal binding window in autism spectrum disorders. Exp Brain Res. (2010) 203:381–9. doi: 10.1007/s00221-010-2240-4 PMC2871100 20390256

[16] van Wassenhove V Grant KW Poeppel D. Temporal window of integration in auditory-visual speech perception. Neuropsychologia. (2007) 45:598–607. doi: 10.1016/j.neuropsychologia.2006.01.001 16530232

[17] Wallace MT Stevenson RA. The construct of the multisensory temporal binding window and its dysregulation in developmental disabilities. Neuropsychologia. (2014) 64:105–23. doi: 10.1016/j.neuropsychologia.2014.08.005 PMC4326640 25128432

[18] Zhou HY Cheung EF Chan RC. Audiovisual temporal integration: Cognitive processing, neural mechanisms, developmental trajectory and potential interventions. Neuropsychologia. (2020) 140:107396. doi: 10.1016/j.neuropsycoogia.2020.107396 32087206

[19] Brandwein AB Foxe JJ Butler JS Russo NN Altschuler TS Gomes H. The development of multisensory integration in high-functioning autism: high-density electrical mapping and psychophysical measures reveal impairments in the processing of audiovisual inputs. Cereb Cortex. (2013) 23:1329–41. doi: 10.1093/cercor/bhs109 PMC3643715 22628458

[20] Kwakye LD Foss-Feig JH Cascio CJ Stone WL Wallace MT. Altered auditory and multisensory temporal processing in autism spectrum disorders. Front Integr Neurosci. (2011) 4:129. 21258617 10.3389/fnint.2010.00129 PMC3024004

[21] World Health Organization. *ICD-11: International classification of diseases* (11th revision) (2019). Available online at: https://icd.who.int/.

[22] Keefe RS Harvey PD. Cognitive impairment in schizophrenia. In: Geyer MA Gross G, editors. Handbook of Experimental Pharmacology, vol. 213. Springer, Heidelberg (2012). p. 11–37. doi: 10.1007/978-3-642-25758-2_2 23027411

[23] Mesholam-Gately RI Giuliano AJ Goff KP Faraone SV Seidman LJ. Neurocognition in first-episode schizophrenia: a meta-analytic review. Neuropsychology. (2009) 23:315. doi: 10.1037/a0014708 19413446

[24] Zhang H Wang Y Hu Y Zhu Y Zhang T Wang J. Meta-analysis of cognitive function in Chinese first-episode schizophrenia: MATRICS Consensus Cognitive Battery (MCCB) profile of impairment. Gen Psychiatry. (2019) 32. doi: 10.1136/gpsych-2018-100043 PMC6677937 31423473

[25] Pinkham AE Hopfinger JB Pelphrey KA Piven J Penn DL. Neural bases for impaired social cognition in schizophrenia and autism spectrum disorders. Schizophr Res. (2008) 99:164–75. doi: 10.1016/j.schres.2007.10.024 PMC2740744 18053686

[26] American Psychiatric Association. Diagnostic and statistical manual of mental disorders. DSM 5 (5th edn.). Washington DC: American Psychiatric Publication (2013).

[27] Opoku-Baah C Schoenhaut AM Vassall SG Tovar DA Ramachandran R Wallace MT. Visual influences on auditory behavioral, neural, and perceptual processes: a review. J Assoc Res Otolaryngol. (2021) 22:365–86. doi: 10.1007/s10162-021-00789-0 PMC8329114 34014416

[28] Surguladze SA Calvert GA Brammer MJ Campbell R Bullmore ET Giampietro V. Audio–visual speech perception in schizophrenia: an fMRI study. Psychiatry Research: Neuroimaging. (2001) 106:1–14. doi: 10.1016/S0925-4927(00)00081-0 11231095

[29] Cook J Barbalat G Blakemore S-J. Top-down modulation of the perception of other people in schizophrenia and autism. Front Hum Neurosci. (2012) 6:175. doi: 10.3389/fnhum.2012.00175 22715325 PMC3375615

[30] Davis J Moylan S Harvey BH Maes M Berk M. Neuroprogression in schizophrenia: pathways underpinning clinical staging and therapeutic corollaries. Aust New Z J Psychiatry. (2014) 48:512–29. doi: 10.1177/0004867414533012 24803587

[31] Capa RL Duval CZ Blaison D Giersch A. Patients with schizophrenia selectively impaired in temporal order judgments. Schizophr Res. (2014) 156:51–5. doi: 10.1016/j.schres.2014.04.001 24768441

[32] Giersch A Lalanne L Corves C Seubert J Shi Z Foucher J. Extended visual simultaneity thresholds in patients with schizophrenia. Schizophr Bullitin. (2009) 35:816–25. doi: 10.1093/schbul/sbn016 PMC2696372 18359954

[33] de Gelder B Vroomen J Annen L Masthof E Hodiamont P. Audio-visual integration in schizophrenia. Schizophr Res. (2003) 59:211–8. doi: 10.1016/S0920-9964(01)00344-9 12414077

[34] Foucher JR Lacambre M Pham BT Giersch A Elliott MA. Low time re solution in schizophrenia. Lengthened windows of simultaneity for visual, auditory and bimodal stimuli. Schizophr Res. (2007) 97:118–27. doi: 10.1016/j.schres.2007.08.013 17884350

[35] Cascio CJ Simon DM Bryant LK DiCarlo G Wallace MT. Neurodevelopmental and neuropsychiatric disorders affecting multisensory processes. Multisensory Percept. (2020), 371–99. doi: 10.1016/B978-012-812492-5.000176

[36] Szycik GR Münte TF Dillo W Mohammadi B Samii A Emrich HM. Audiovisual integration of speech is disturbed in schizophrenia: an fMRI study. Schizophr Res. (2009) 110:111–8. doi: 10.1016/j.schres.2009.03.003 19303257

[37] Zhou HY Cai XL Weigl M Bang P Cheung EF Chan RC. Multisensory temporal binding window in autism spectrum disorders and schizophrenia spectrum disorders: A systematic review and meta analysis. Neurosci Biobehav Rev. (2018) 86:66–76. doi: 10.1016/j.neubiorev.2017.12.013 29317216

[38] Zhou HY Cui XL Yang BR Shi LJ Luo XR Cheung EF. Audiovisual temporal processing in children and adolescents with schizophrenia and children and adolescents with autism: evidence from simultaneity judgment tasks and eye-tracking data. Clin psychol Sci. (2022) 10:482–498. doi: 10.1177/21677026211031543

[39] Zhou HY Lai IY Hung KS Chan MK Ho ZT Lam JP. Audiovisual temporal processing in adult patients with first-episode schizophrenia and high-functioning autism. Schizophrenia. (2022) 8:75. doi: 10.1038/s41537-022-00284-2 36138029 PMC9500036

[40] Johannes S Münte TF Heinze HJ Mangun GR. Luminance and spatial attention effects on early visual processing. Cogn Brain Res. (1995) 2:189–205. doi: 10.1016/0926-6410(95)90008-X 7580401

[41] Key APF Dove GO Maguire MJ. Linking brainwaves to the brain: an ERP primer. Dev Neuropsychol. (2005) 27:183–215. doi: 10.1207/s15326942dn2702_1 15753046

[42] Roa Romero Y Keil J Balz J Niedeggen M Gallinat J Senkowski D. Alpha-band oscillations reflect altered multisensory processing of the McGurk illusion in schizophrenia. Front Hum Neurosci. (2016) 10:41. doi: 10.3389/fnhum.2016.00041 26903845 PMC4751891

[43] Baart M Stekelenburg JJ Vroomen J. Electrophysiological evidence for speech-specific audiovisual integration. Neuropsychologia. (2014) 53:115–21. doi: 10.1016/j.neuropsychologia.2013.11.011 24291340

[44] Besle J Fischer C Bidet-Caulet A Lecaignard F Bertrand O Giard MH. Visual activation and audiovisual interactions in the auditory cortex during speech perception: intracranial recordings in humans. J Neurosci. (2008) 28:14301–10. doi: 10.1523/JNEUROSCI.2875-08.2008 PMC6671467 19109511

[45] Ghaneirad E Saenger E Szycik GR Čuš A Moöde L Sinke C. Deficient audiovisual speech perception in schizophrenia: an ERP study. Brain Sci. (2023) 13:970. doi: 10.3390/brainsci13060970 37371448 PMC10295846

[46] Wynn JK Jahshan C Altshuler LL Glahn DC Green MF. Event-related potential examination of facial affect processing in bipolar disorder and schizophrenia. psychol Med. (2013) 43:109–17. doi: 10.1017/S0033291712001006 PMC3959981 22583955

[47] Gröhn C Norgren E Eriksson L. A systematic review of the neural correlates of multisensory integration in schizophrenia. Schizophr Research: Cogn. (2022) 27:100219. doi: 10.1016/j.scog.2021.100219 PMC8502765 34660211

[48] Stekelenburg JJ Maes JP van Gool AR Sitskoorn M Vroomen J. Deficient multisensory integration in schizophrenia: An event-related potential study. Schizophr Res. (2013) 147:253–61. doi: 10.1016/j.schres.2013.04.038 23707640

[49] Balz J Roa Romero Y Keil J Krebber M Niedeggen M Gallinat J. Beta/gamma oscillations and event-related potentials indicate aberrant multisensory processing in schizophrenia. Front Psychol. (2016) 7:1896. doi: 10.3389/fpsyg.2016.01896 27999553 PMC5138197

[50] Liu T Pinheiro AP Zhao Z Nestor PG McCarley RW Niznikiewicz M. Simultaneous face and voice processing in schizophrenia. Behav Brain Res. (2016) 305:76–86. doi: 10.1016/j.bbr.2016.01.039 26804362

[51] Chen YC Shore DI Lewis TL Maurer D. The development of the perception of audiovisual simultaneity. J Exp Child Psychol. (2016) 146:17–33. doi: 10.1016/j.jecp.2016.01.010 26897264

[52] Zampini M Guest S Shore DI Spence C. Audio-visual simultaneity judgments. Percept Psychophysics. (2005) 67:531–44. doi: 10.3758/BF03193329 16119399

[53] Arrighi R Alais D Burr D. Perceptual synchrony of audiovisual streams for natural and artificial motion sequences. J Visualized Experiments. (2006) 6:260–8. doi: 10.1167/6.3.6 16643094

[54] Pöppel E Artin TT. Mindworks: Time and conscious experience. San Diego: Harcourt Brace Jovanovich (1988).

[55] van Eijk RLJ Kohlrausch A Juola JF van de Par S. Audiovisual synchrony and temporal order judgments: Effects of experimental method and stimulus type. Percept Psychophysics. (2008) 70:955–68. doi: 10.3758/PP.70.6.955 18717383

[56] DSM-IV-TR, APA. Diagnostic and statistical manual of mental disorders. Washington, DC: American Psychiatric Association (2000). pp. 70–6.

[57] Kay SR Fiszbein A Opler LA. The positive and negative syndrome scale (PANSS) for schizophrenia. Schizophr Bull. (1987) 13:261–76. doi: 10.1093/schbul/13.2.261 3616518

[58] Gerhold M Huss M Luecke M. PANSS - Deutsche Übersetzung von Interviewleitfaden, Beurteilungskriterien und Profilform. (1999). Available online at: http://www.mhs.com (accessed May 22, 2024).

[59] Lehrl S. Mehrfachwahl-Wortschatz-Intelligenztest. MWT-B. Spitta, Bahlingen (1995).

[60] Lee TW Lewicki MS Sejnowski TJ. ICA mixture models for unsupervised classification and automatic context switching. Proc Int Workshop Independent Component Anal. (1999), 209–14.

[61] Näätänen R Picton T. The N1 wave of the human electric and magnetic response to sound: a review and an analysis of the component structure. Psychophysiology. (1987) 24:375–425. doi: 10.1111/j.14698986.1987.tb00311.x 3615753

[62] Noel JP de Niear M van der Burg E Wallace MT. Audiovisual simultaneity judgment and rapid recalibration throughout the lifespan. PloS One. (2016) 11:e0161698. doi: 10.1371/journal.pone.0161698 27551918 PMC4994953

[63] Kaganovich N Schumaker J. Electrophysiological correlates of individual differences in perception of audiovisual temporal asynchrony. Neuropsychologia. (2016) 86:119–30. doi: 10.1016/j.neuropsychologia.2016.04.015 PMC5137199 27094850

[64] Cohen J Cohen P West SG Aiken LS. Applied Multiple Regression/Correlation Analysis for the Behavioral Sciences. London: Routledge (2013).

[65] Borgolte A Bransi A Seifert J Toto S Szycik GR Sinke C. Audiovisual simultaneity judgements in synaesthesia. Multisensory Res. (2021) 34:681–92. doi: 10.1163/22134808-bja10050 33984831

[66] Matthews N Welch L Achtman R Fenton R FitzGerald B. Simultaneity and temporal order judgments exhibit distinct reaction times and training effects. PloS One. (2016) 11:e0145926. doi: 10.1371/journal.pone.0145926 26756716 PMC4710527

[67] Basharat A Adams MS Staines WR Barnett-Cowan M. Simultaneity and temporal order judgments are coded differently and change with age: an event-related potential study. Front Integr Neurosci. (2018) 12:15. doi: 10.3389/fnint.2018.00015 29755327 PMC5932149

[68] Haß K Sinke C Reese T Roy M Wiswede D Dillo W. Enlarged temporal integration window in schizophrenia indicated by the double-flash illusion. Cogn neuropsychiatry. (2017) 22:145–58. doi: 10.1080/13546805.2017.1287693 28253091

[69] Sterzer P Adams RA Fletcher P Frith C Lawrie SM Muckli L. The predictive coding account of psychosis. Biol Psychiatry. (2018) 84:634–43. doi: 10.1016/j.biopsych.2018.05.015 PMC6169400 30007575

[70] Sterzer P Voss M Schlagenhauf F Heinz A. Decision-making in schizophrenia: a predictive-coding perspective. NeuroImage. (2019) 190:133–43. doi: 10.1016/j.neuroimage.2018.05.074 29860087

[71] Rao RP Ballard DH. Predictive coding in the visual cortex: a functional interpretation of some extra-classical receptive-field effects. Nat Neurosci. (1999) 2:79–87. doi: 10.1038/4580 10195184

[72] Friston K Kiebel S. Predictive coding under the free-energy principle. Philos Trans R Soc B Biol Sci. (2009) 364:1211–21. doi: 10.1098/rstb.2008.0300 PMC2666703 19528002

[73] Bendixen A Scharinger M Strauß A Obleser J. Prediction in the service of comprehension: modulated early brain responses to omitted speech segments. Cortex. (2014) 53:9–26. doi: 10.1016/j.cortex.2014.01.001 24561233

[74] Peelle JE Davis MH. Neural oscillations carry speech rhythm through to comprehension. Front Psychol. (2012) 3:320. doi: 10.3389/fpsyg.2012.00320 22973251 PMC3434440

[75] Sohoglu E Peelle JE Carlyon RP Davis MH. Predictive top-down integration of prior knowledge during speech perception. J Neurosci. (2012) 32:8443–53. doi: 10.1523/JNEUROSCI.5069-11.2012 PMC6620994 22723684

[76] van Wassenhove V. Speech through ears and eyes: interfacing the senses with the supramodal brain. Front Psychol. (2013) 4:388. doi: 10.3389/fpsyg.2013.00388 23874309 PMC3709159

[77] van Wassenhove V Grant KW Poeppel D. Visual speech speeds up the neural processing of auditory speech. Proc Natl Acad Sci USA. (2005) 102:1181–6. 10.1073/pnas.0408949102 PMC545853 15647358

[78] Stekelenburg JJ Vroomen J. Neural correlates of multisensory integration of ecologically valid audiovisual events. J Cogn Neurosci. (2007) 19:1964–73. doi: 10.1162/jocn.2007.19.12.1964 17892381

[79] Luck SJ. An Introduction to the Event-Related Potential Technique. 2nd ed. USA: MIT Press (2014).

[80] Vogel EK Luck SJ Shapiro KL. Electrophysiological evidence for a postperceptual locus of suppression during the attentional blink. J Exp Psychology: Hum Percept Perform. (1998) 24:1656. doi: 10.1037/0096-1523.24.6.1656 9861716

[81] Stekelenburg JJ Vroomen J. An event-related potential investigation of the time-course of temporal ventriloquism. Neuroreport. (2005) 16(6):641–4. 10.1097/00001756-200504250-00025 15812324

[82] Binder M. An ERP study of audiovisual simultaneity perception. Seeing Perceiving. (2012) 25:159–9. doi: 10.1163/187847612X647900

[83] Behne DM Sorati M Alm M. Perceived Audiovisual Simultaneity in Speech by Musicians and Nonmusicians: Preliminary Behavioral and Event-Related Potential (ERP) Findings. Stockholm: AVSP (2017) p. 100–4.

[84] Horsfall R Harrison N Meyer G Wuerger S. Perceived audio-visual simultaneity is recalibrated by the visual intensity of the preceding trial. Multisensory Res. (2024) 37:143–62. doi: 10.1163/22134808-bja10121 38714315

